# Tact in Sanatorium Management

**Published:** 1918-03-02

**Authors:** Florence Lloyd

**Affiliations:** Sister


### THE EDITOR'S LETTER-BOX.

### Tact in Sanatorium Management.

*To the Editor of* The Hospital.

Sir,—Your remarks about the complaints of sanatorium patients in your issue of February 2 brought to my mind a series of complaints from the patients of a large sanatorium a few years ago which led to a public inquiry. The medical officer, in the course of his evidence, said the eggs were not bad, but they might have been "musty." Now, as he was that most wonderful being, a *male* medical superintendent, why did he not, in a few "well-chosen words," persuade the patients that the eggs were new-laid, and so clear the atmosphere and avoid the inquiry? There are several things required to make sanatorium life a happy one for all concerned. One of the chief things required is tact, and we venture to claim for women medical superintendents a fair amount of this valuable article.—Yours faithfully,

February 10, 1918. Florence Lloyd, Sister.

